# Off-clamp partial nephrectomy has a positive impact on short- and long-term renal function: a systematic review and meta-analysis

**DOI:** 10.1186/s12882-018-0993-3

**Published:** 2018-07-31

**Authors:** Wen Deng, Xiaoqiang Liu, Jieping Hu, Luyao Chen, Bin Fu

**Affiliations:** Department of Urology, First Affiliated Hospital of Nanchang University, 17 Yongwai Street, Nanchang City, Jiangxi Province China

**Keywords:** Off-clamp, Partial nephrectomy, Renal function, Short-term, Long-term, eGFR, Creatinine

## Abstract

**Background:**

Ongoing efforts are focused on shortening ischemia intervals as much as possible during partial nephrectomy to preserve renal function. Off-clamp partial nephrectomy (off-PN) has been a common strategy for to avoid ischemia in small renal tumors. Although studies comparing the advantages between off-PN with conventional on-clamp partial nephrectomy (on-PN) have been reported, the impact on short- and especially long-term renal function of the two surgical methods has not been discussed seriously and remained unclear. Our purpose is to evaluate the impact on short- (within postoperative 3 months) and long-term (postoperative 6 months or longer) renal function of off-PN compared with that of on-PN.

**Methods:**

We comprehensively searched databases, including PubMed, EMBASE, and the Cochrane Library, without restrictions on language or region. A systematic review and cumulative meta-analysis of the included studies were performed to assess the impact of the two techniques on short- and long-term renal function.

**Results:**

A total of 23 retrospective studies and 2 prospective cohort studies were included. The pooled postoperative short-term decrease of estimated glomerular filtration rate (eGFR) was significantly less in the off-PN group (weighted mean difference [WMD]: 4.81 ml/min/1.73 m^2^; 95% confidence interval [CI]: 3.53 to 6.08; *p* < 0.00001). The short-term increase in creatinine (Cr) level in the on-PN group was also significant (WMD: − 0.05 mg/dl; 95%CI: − 0.09 to − 0.00; *p* = 0.04). Significant differences between groups was observed for the long-term change and percent (%) change of eGFR (*p* = 0.04 and *p* < 0.00001, respectively) but not for long-term Cr change (*p* = 0.40). The postoperative short-term eGFR and Cr levels, but not the postoperative long-term eGFR, differed significantly between the two groups. The pooled odds ratios for acute renal failure and postoperative progress to chronic kidney disease (stage≥3) in the off-PN group were found to be 0.25 (*p* = 0.003) and 0.73 (*p* = 0.34), respectively, compared with the on-PN group.

**Conclusions:**

Off-PN exerts a positive impact on the short- and long-term renal function compared with conventional on-PN. Given the inherent limitations of our included studies, large-volume and well-designed RCTS with extensive follow up are needed to confirm and update the conclusion of this analysis.

**Electronic supplementary material:**

The online version of this article (10.1186/s12882-018-0993-3) contains supplementary material, which is available to authorized users.

## Background

Scholars agree that the partial nephrectomy for small renal mass is advantageous over radical nephrectomy in terms of renal function [1, 2]. The conventional partial nephrectomy technique includes the clamping of the renal artery (on-PN); this method allows tumor resection and renal reconstruction in a relatively bloodless field [3–5]. However, occluding the renal artery places the remaining nephrons at risk of ischemia−reperfusion injury and mitigates the renoprotective purpose of surgery [3, 6]. Shorter ischemia intervals have been correlated with better renal functional preservation [7, 8]. Off-clamp partial nephrectomy (off-PN) has been a common strategy to avoid ischemia in small renal tumor. Although a consensus has been reached on off-PN risking more blood loss, the impact of the two methods on the change of postoperative short- and long-term renal function remains unclear [1, 9]. Therefore, we systematically searched and analyzed the clinical studies comparing off-PN with on-PN for small renal masses published until January 2018 to assess the methods’ impact on short- and long-term renal function.

## Methods

The literature search methods, inclusion and exclusion criteria, outcome measures, and statistical analysis methods were well defined in a prospective protocol.

### Literature-search strategy

A literature search was performed in January 2018 with no restriction to region or language. The primary sources were the electronic databases of PubMed, EMBASE, and the Cochrane Library. The following terms and their combinations were searched as follows: (“partial nephrectomy” OR “nephron sparing surgery” [Title/Abstract]) and (“clamp*” OR “ischemia” [Title/Abstract]). Our computer search was supplemented with manual searches of reference lists of all retrieved review articles. When multiple studies were reported by the same institution and/or authors, the most complete report was included in our analysis.

### Inclusion and exclusion criteria

All retrospective or prospective comparative studies (cohort or case−control studies) containing a comparison of off-PN and on-PN with or without a third group, such as cold-ischemia partial nephrectomy, and those providing available data to assess postoperative renal function, were included. Animal experimental studies, editorials, letters to the editor, review articles, case reports, conference abstracts, studies without available data about postoperative renal function, and non-comparative studies were excluded.

### Data extraction and outcome measures

Two of the authors extracted and summarized data from the included studies independently. Any disagreement was resolved by mutual discussion with another two adjudicating senior authors.

The primary outcomes were short-term change of estimated glomerular filtration rate (eGFR), short-term change of Cr level, long-term change of eGFR, long-term % change of eGFR, and long-term change of Cr level.

The secondary outcomes were postoperative long-term eGFR, short-term eGFR, short-term Cr level, postoperative increase in CKD (stage≥3), and postoperative acute renal failure (ARF).

### Quality assessment and statistical analysis

The modified Newcastle−Ottawa scale [10, 11] was used to assess the methodological quality of nonrandomized studies comprising three factors: patient selection, comparability of study groups, and assessment of outcome. Every study was scored from 0 to 9.

The level of evidence was assessed on the basis of the criteria enacted by the Center for Evidence-Based Medicine in Oxford used to rate the included studies [12].

Review Manager 5.3 (Cochrane Collaboration, Oxford, UK) was used to perform all meta-analysis. The odds ratio (OR) with 95% confidence interval (CI) was used to compare the dichotomous variables consisting of ARF and postoperative increase in chronic kidney disease (CKD; stage≥3), and the weighted mean difference (WMD) with 95%CI was used to compare the remaining continuous variables. The corresponding authors were contacted when the data were missing or incomplete. The technique summarized by Hozo et al. was used to convert medians to means [13].

Statistical heterogeneity was considered significant when the Cochrane Q test *p* value was < 0.10. The standard heterogeneity test, I^2^ statistic, was used to assess the consistency of the effect sizes. The fixed-effects model was used when no significant heterogeneity exists between the studies; otherwise, the random-effects model is used [14].

Subgroup analysis was performed to verify the impact of two surgical procedures and to assess the efficacy of different studies in more homogeneous subsets in accordance with the sample size in the studies.

Sensitivity analysis was performed by repeating the primary analysis without including the highest scored study or studies when some papers achieved the highest scores at the same time.

STATA SE 12.0 was then utilized to evaluate potential publication bias, which was screened on funnel plots and assessed statistically using the Begg’s test and Egger’s test. The tests were two sided, and the *p* values of < 0.05 were considered significant statistically.

## Results

**Fig. 1:**
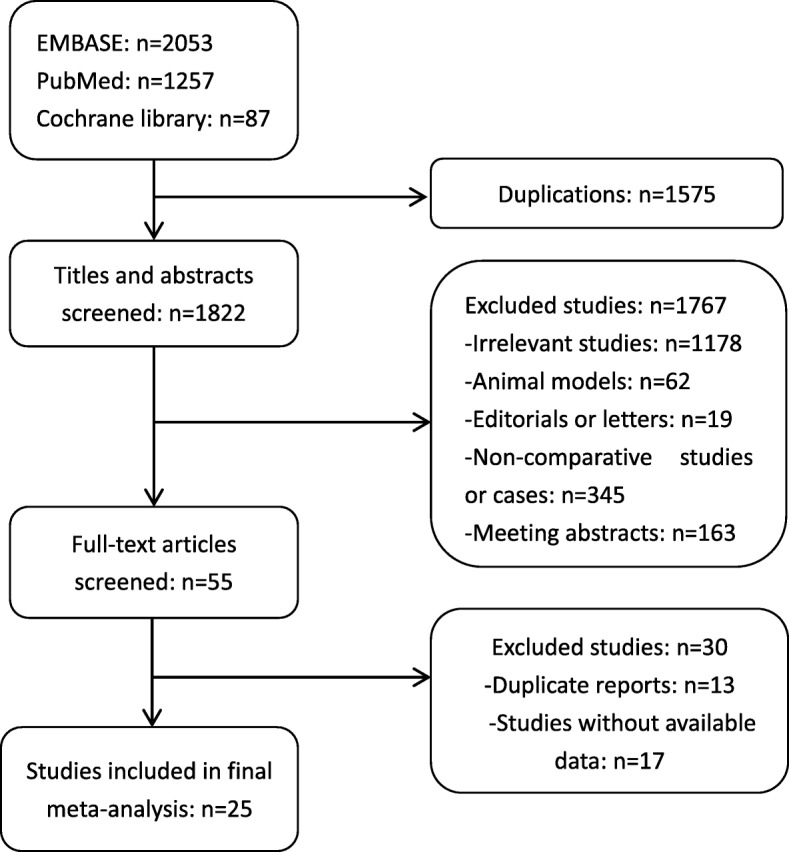
Flowchart of study selection

A total of 3766 patients (off-PN 1197; on-PN 2569) from 25 studies fulfilled the predefined inclusion criteria and were included in this analysis. The detailed process of research screening and selection is shown in Fig. 1. Most eligible studies were designed retrospectively; among these works, 7 included a third group besides the off-PN and on-PN groups. The comparison of preoperative renal function between off-PN and on-PN was not significantly different in 13 studies, whereas that in others differed significantly or did not use statistics between the two groups.

### Characteristics of included studies

Table 1 summarizes the characteristics of the included studies [9, 15–38]. Among eligible studies, two were prospective cohort studies (level of evidence: 2b) [28, 35], 19 were retrospective studies comparing contemporary series of patients (level of evidence: 3b) [9, 15, 16, 18–21, 23, 25, 27, 29–34, 36–38], and the remaining 4 studies used a historical series as controls (level of evidence: 4) [17, 22, 24, 26]. A total of 14 studies containing a total number of ≤110 patients were considered as small-sample studies [9, 15–20, 23, 28, 29, 34–36, 38], whereas those containing over 110 patients in all were considered as large-sample studies [21, 22, 24–27, 30–33, 37].

**Table 1:** Characteristics of included studies

Study	Design	Country	Age, mean		Patients, no			Surgical approach	Tumor Size^a^		Follow-up off/on	Level of evidence	Quality Score
			off	on	off	on	total		off	on(cm)			
Guillonneau^2003^ [15]	R	France	60.6	60.0	12	16	28	LPN	1.9	2.5	12.2/1.21 m	3b	6
Kane^2004^ [16]	R	USA	62	52	12	15	27	LPN	2.2	3.0	4.3/4.3 m	3b	6
Kobayashi^2008^ [17]	R	Japan	60.3	51.8	5	5	10	LPN	2.4	2.0	1/1 m	4	5
Hong^2009^ [19]	R	Korea	55.5	54.8	17	16	33	LPN	2.1	3.3	26.1/31.3 m	3b	6
Koo^2010^ [20]	R	Korea	58.0	63.7	11	10	21	LPN	2.6	2.3	postoperati	3b	5
Thompson^2010^ [21]	R	USA	63	62	96	362	458	OPN,LPN	2.5	3.4	39.6/39.6 m	3b	5
Smith^2011^ [22]	R	USA	62	62	192	116	308	O + L + R	3.0	2.8	12/12 m	4	5
Petrasz^2012^ [23]	R	Poland	54.7	58.3	13	25	38	LPN	3.1	3.3	postoperative	3b	4
George^2013^ [25]	R	USA	59.2	59.4	150	289	439	LPN	2.7	3.3	6/6 m	3b	6
Kaczmarek^2013^ [27]	RM	USA	60.4	60.2	49	283	332	RPN	NA		21/21 m	3b	6
Porpiglia^2012^ [24]	R	Italy	65.1	61.3	41	76	117	LPN	2.4	3.2	postoperative	4	7
Salevitz^2015^ [33]	R	USA	67	62	95	191	286	O + L + R	2.5	2.9	50.9/32.2 m	3b	5
Ener^2016^ [34]	RM	Turkey	53.0	54.4	12	22	34	RPN	3.3	3.2	3/3 m	3b	6
Wang^2016^ [35]	P	China	54.4	54.4	22	22	44	LPN	2.0	1.9	0.2/0.2 m	2b	8
Anderson^2017^ [36]	R	USA	58.5	56.1	50	50	100	RPN	3.1	3.6	9/9 m	3b	6
Rosen^2017^ [37]	RM	USA	60.0	61.5	41	82	123	RPN	1.8	2.0	9.2/9.2 m	3b	8
Verze^2017^ [38]	R	Italy	56.0	57.2	64	37	101	LPN	4.7	5.1	6/6 m	3b	7
Weizer^2008^ [18]	R	USA	57	53	36	25	61	LPN	1.9	1.9	10/12 m	3b	4
Hung^2013^ [26]	R	USA	NA		81	272	353	LPN,RPN	3.4	2.9	postoperative	4	4
Lee^2014^ [31]	R	Korea	53.6	54.5	39	201	240	OPN	2.0	2.3	12/12 m	3b	4
Komninos^2015^ [32]	R	Korea	53	51	23	114	137	RPN	1.7	3.3	12/12 m	3b	4
T Tawatchi^2018^ [9]	R	Thailand	50.1	56.0	12	27	39	RPN	2.2	3.5	26/19 m	3b	4
Acar^2014^ [29]	R	Turkey	51.1	46.2	30	14	44	RPN	3.8	3.6	18.9/18.9 m	3b	7
Krane^2013^ [28]	p	USA	57.1	62.4	19	58	77	RPN	1.6	2.9	4.5/5.5 m	2b	4
Jabaji^2014^ [30]	R	USA	55	57	75	241	316	OPN	3.9	3.3	34.7/34.7 m	3b	4

off = off-clamp partial nephrectomy; on = on-clamp partial nephrectomy; LPN = laparoscopic partial nephrectomy; OPN = open partial nephrectomy; RPN = robot-assisted partial nephrectomy; O + L + R = OPN + LPN + RPN; m = months

*R* retrospective, *P* prospective, *RM* respective matched, *NA* data not available

^a^mean tumor size

### Quality of included studies

No appropriate protocol was employed in advance to allocate patients and assign treatment in retrospective studies but only based on physician’s discretion. Information on allocation concealment or blinding method was not mentioned in studies. Patients were sequentially distributed individually for off-PN or on-PN by the same surgeon in two prospective studies [28, 35]. A modified Newcastle−Ottawa scale was used to evaluate the risks of bias (Additional file 1). Two prospective studies achieved a proper protocol for the design. The matching about preoperative characteristics was performed in terms of age, tumor size, preoperative renal function, American Society of Anesthesiologists score, gender, tumor side, body mass index, nephrectomy (R.E.N.A.L.) score, and tumor location. Outcomes include the assessment of renal function in the postoperative period. Methods for handling missing data and intention-to-treat analysis were not adequately described in some studies.

### Primary outcomes

**Fig. 2:**
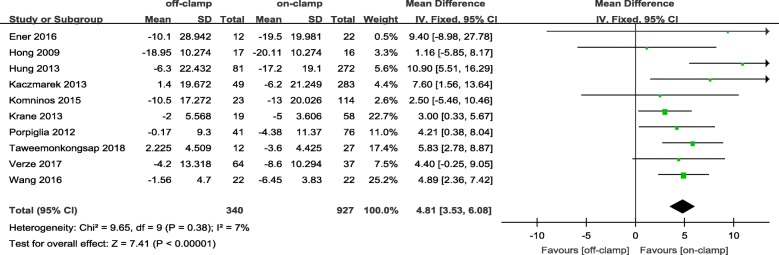
Forest plot and meta-analysis of postoperative short-term eGFR change

**Fig. 3:**
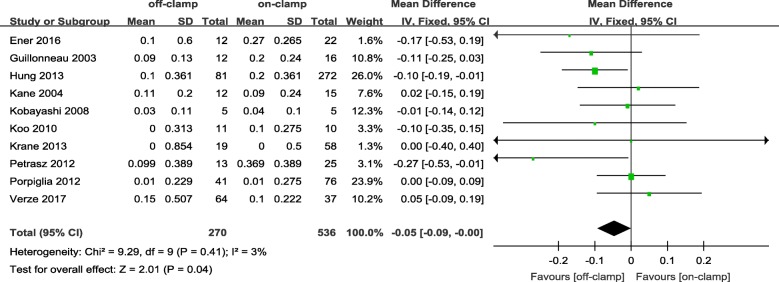
Forest plot and meta-analysis of postoperative short-term Cr level change

**Fig. 4:**
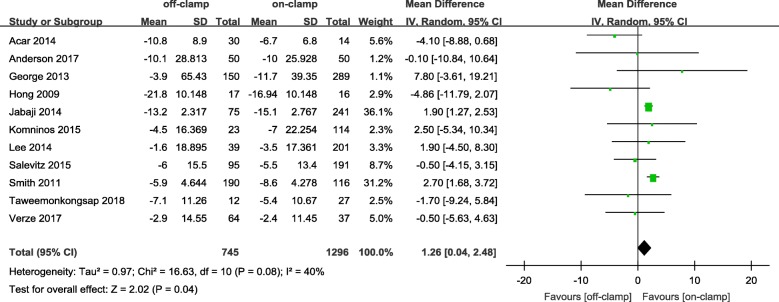
Forest plot and meta-analysis of postoperative long-term eGFR change

**Fig. 5:**
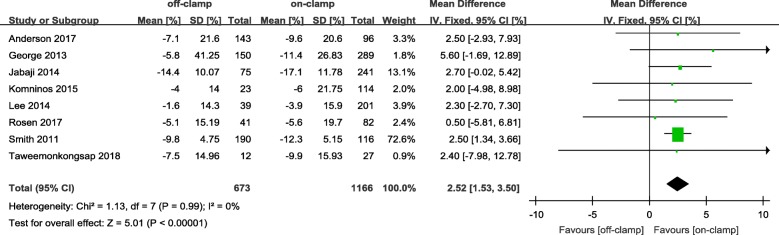
Forest plot and meta-analysis of percent change of eGFR

**Fig. 6:**
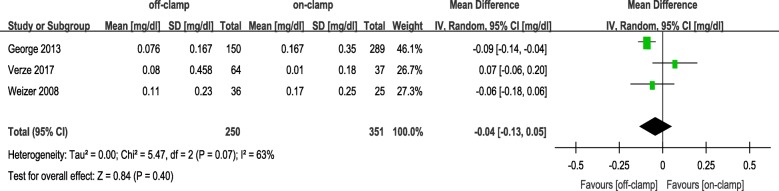
Forest plot and meta-analysis of postoperative long-term Cr level change

Within the postoperative 3 months, the short-term decrease of eGFR and short-term increase of Cr level in an off-PN group were significantly less ([WMD: 4.81; 95%CI: 3.53 to 6.08; *p* < 0.00001] and [WMD: − 0.05; 95%CI: − 0.09 to − 0.00; *p* = 0.04], respectively) (Figs. 2 and 3). After 6 months or more of surgery, the long-term loss of eGFR and percent decrease of eGFR in the off-PN group remained significantly less than that of the on-PN group ([WMD: 1.26; 95%CI: 0.04 to 2.48; *p* = 0.04] and [WMD: 2.52; 95%CI: 1.53 to 3.50; *p* < 0.00001], respectively) (Figs. 4 and 5). However, the long-term increase of Cr level was insignificant in an on-PN group (WMD: − 0.04; 95%CI: − 0.13 to 0.05; *p* = 0.40) (Fig. 6).

### Secondary outcomes

Within postoperative 3 months, the short-term eGFR was significantly higher in an off-PN group (WMD: 9.72; 95%CI: 6.13 to 13.30; *p* < 0.00001) (Additional file 2). On the contrary, the short-term Cr level was significantly lower in the off-PN group (WMD: − 0.08; 95%CI: − 0.13 to − 0.02; *p* = 0.007) (Additional file 3). After 6 months or more of surgery, the long-term eGFR was not significantly different between the two groups (WMD: − 0.18; 95%CI: − 3.89 to 3.53; *p* = 0.92) (Additional file 4). Postoperative ARF was more likely to occur in the on-PN group (OR: 0.25; 95%CI: 0.10 to 0.63; *p* = 0.003) (Additional file 5) than in the off-PN group. The morbidity of CKD (stage≥3) in the postoperative period was not significantly different between the two groups (OR: 0.73; 95%CI: 0.39 to 1.39; *p* = 0.34) (Additional file 6) (Table 2).

**Table 2:** Results of meta-analysis comparison of off-clamp partial nephrectomy and on-clamp partial nephrectomy

Outcomes of interest	Studies, no	off-PN patients, no	on-PN patient, no	WMD/OR (95%CI)	*p* value	Study heterogeneity			
						Chi^2^	df	*p* value	I^2^,%
Primary outcomes									
short-term change of eGFR	10	340	927	4.81(3.53,6.08)	< 0.00001	9.65	9	0.38	7
short-term change of Cr level	10	270	536	−0.05(− 0.09,-0.00)	0.04	9.29	9	0.41	3
long-term change of eGFR	11	745	1296	1.26(0.04,2.48)	0.04	16.63	10	0.08	40
long-term % change of eGFR	8	673	1166	2.52(1.53,3.50)	< 0.00001	1.13	7	0.99	0
long-term change of Cr level	3	250	351	−0.04(−0.13, 0.05)	0.40	5.47	2	0.07	63
Secondary outcomes									
long-term eGFR	9	582	1040	−0.18(−3.89,3.53)	0.92	37.54	8	< 0.00001	79
short-term eGFR	9	327	915	9.72(6.13,13.30)	< 0.00001	43.70	8	< 0.00001	82
short-term Cr level	7	237	452	−0.08(−0.13,-0.02)	0.007	2.23	6	0.90	0
increase in CKD(stage≥3)	5	170	522	0.73(0.39,1.39)^a^	0.34	0.97	4	0.91	0
postoperative AFR	3	224	568	0.25(0.10,0.63)^a^	0.003	0.10	2	0.95	0

off-PN = off-clamp partial nephrectomy; on-PN = on-clamp partial nephrectomy; WMD/OR = weighted mean difference/odds ratio; df = degrees of freedom; CI = confidence interval; eGFR = estimated glomerular filtration rate; Cr = serum creatinine

*CKD* chronic kidney disease, *AFR* acute renal failure

^a^Odds ratio

### Subgroup analysis

Subgroup analysis was performed to evaluate whether the primary outcomes were different in accordance with the sample size (Additional files 7, 8, 9, 10 and 11) (Table 3).

**Table 3:** Subgroup analysis and sensitivity analysis of primary outcomes

Comparison	MD (95%CI)	*p* value	Study heterogeneity			
			Chi^2^	df	*p* value	I^2^,%
short-term change of eGFR	4.81(3.53,6.08)	< 0.00001	9.65	9	0.38	7
Studies (LS)	6.24(3.62,8.86)	< 0.00001	5.00	3	0.17	40
Studies (SS)	4.36(2.91,5.82)	< 0.00001	3.14	5	0.68	0
Exclusion of [35]	4.78(3.31,6.25)	< 0.00001	9.65	8	0.29	17
short-term change of Cr level	−0.05(−0.09,-0.00)	0.04	9.29	9	0.41	3
Studies (LS)	−0.05(− 0.12,0.01)	0.11	2.29	1	0.13	56
Studies (SS)	−0.04(− 0.11,0.02)	0.20	6.95	7	0.43	0
Exclusion of [24, 38]	−0.08(− 0.14,-0.02)	0.006	5.33	7	0.62	0
long-term change of eGFR	1.26(0.04,2.48)	0.04	16.63	10	0.08	40
Studies (LS)	2.08(1.55,2.61)	< 0.00001	4.63	5	0.46	0
Studies (SS)	−2.57(−5.35, 0.22)	0.07	1.69	4	0.79	0
Exclusion of [29, 38]	2.02(1.49,2.54)	< 0.00001	9.55	8	0.30	16
long-term % change of eGFR	2.45(1.46,3.44)	< 0.00001	2.31	7	0.94	0
Studies (LS)	2.52(1.51,3.52)	< 0.00001	1.13	5	0.95	0
Studies (SS)	−0.36(−6.82,6.10)	0.91	0.44	1	0.51	0
Exclusion of [37]	2.50(1.49,3.50)	< 0.00001	1.93	6	0.93	0
long-term change of Cr level	− 0.04(− 0.13,0.05)	0.40	5.47	2	0.07	
Studies (LS)	−0.09(− 0.14,-0.04)	0.0002	Not applicable			
Studies (SS)	0.00(−0.12,0.13)	0.95	2.08	1	0.15	52
Exclusion of [38]	−0.09(− 0.13,-0.04)	0.0002	0.21	1	0.65	0

*MD* mean difference, *CI* confidence interval, *df* degrees of freedom, *eGFR* estimated glomerular filtration rate, *Cr* serum creatinine, *LS* large sample study, *SS* small sample study

In the large sample size subgroup, a significant difference from the original analysis was obtained in all the primary outcomes except for the short-term change of Cr level.

In the small sample size subgroup, the degree of between-study heterogeneity for all primary outcomes decreased except for the long-term change of Cr level, but the significant difference was no longer found in all primary outcomes except for short-term change of eGFR.

### Sensitivity analysis

Sensitivity analysis was performed by excluding the highest scored study or studies when given the highest score at the same time on the basis of the modified Newcastle−Ottawa Scale. No change in the significance of any of the primary outcomes was noted in the sensitivity analysis. The degree of between-study heterogeneity dropped down to zero for all the primary outcomes except for the short-term eGFR change, of which the degree of between-study heterogeneity slightly increased from 7 to 17% (Table 3).

### Publication bias

**Fig. 7:**
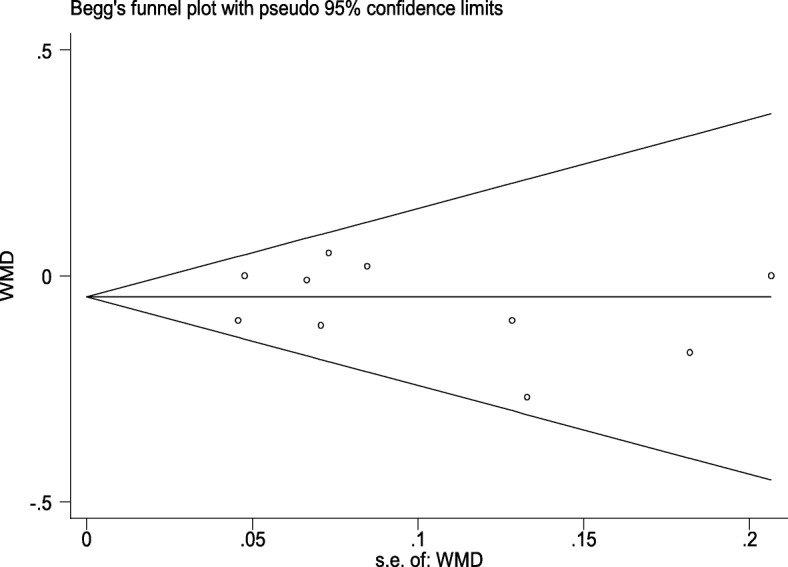
funnel plot of the studies that reported short-term change of Cr level

A funnel plot of the studies that reported the short-term change of Cr level is shown in Fig. 7. All studies lie inside the 95%CI. Begg’s test and Egger’s test were used to evaluate publication bias. There was no significant bias (Begg’s test: *p* = 0.655; Egger’s test: *p* = 0.521).

## Discussion

Nephron-sparing surgery has been the recommended treatment of clinical T1a renal masses and favored over radical nephrectomy in patients with T1b tumors when technically feasible [39]. Every minute counts to preserve renal function when the renal hilum is clamped during partial nephrectomy [7, 40, 41]. For minimizing ischemic injury as much as possible to preserve the functional renal nephron, avoiding ischemia altogether by performing surgery entirely off-clamp is a good strategy. Many studies concluded that partial nephrectomy for small renal masses can be performed without hilar clamping, although considered as a procedure of potentially increased risk of augmented blood loss [2, 3, 5, 9, 15–38]. However, the impact of off-PN on short- and long-term renal functional residual capacity compared with conventional on-PN remains unclear [27]. Thus, we reviewed the published studies and conducted a standard meta-analysis to evaluate the impact of off-PN on short- and long-term renal function compared with conventional on-PN.

Within the postoperative 3 months, the short-term decrease in eGFR was found in both groups in almost all the eligible studies [19, 24, 26, 28, 32, 34, 35, 38], except for Taweemonkongsap et al. [9] and Kaczmarek et al. [27]. The postoperative short-term eGFR of the off-PN group reported by Taweemonkongsap et al. [9] and Kaczmarek et al. [27] achieved an increase compared with preoperative eGFR (mean: 2.225 ml/min/1.73 m^2^ and 1.4 ml/min/1.73 m^2^, respectively). In the two studies, preoperative eGFR records in the off-PN group were higher, and all surgeries were robot assisted, which promoted an enhanced and hastened postoperative renal functional recovery. In all included studies, the short-term decrease of eGFR in the on-PN group was more than that in the off-PN group.

Although no significant difference was found for the postoperative short-term eGFR between two groups in the two studies [24, 35], the pooled analysis of postoperative short-term eGFR showed a significantly higher eGFR in the off-PN group (WMD: 9.72; 95%CI: 6.13 to 13.30; *p* < 0.00001). This result was consistent with a significantly higher decrease of the pooled short-term eGFR in the on-PN group.

The pooled analysis for short-term change of Cr level indicated a significantly higher Cr level increase in the on-PN group compared with that in the off-PN group. While most of the included studies showed a higher increase in the on-PN group [15, 17, 20, 23, 24, 26, 28, 34], the remaining study reported by Kane et al. [16] showed a higher increase, which was probably driven more by a 38% larger tumor size than by the effect of renal artery occlusion in the off-PN group than in the on-PN group. All included studies showed a higher postoperative short-term Cr level in the on-PN group than in the off-PN group in accordance with a pooled meta-analysis for the postoperative short-term Cr level; this result also indicates the better renal functional outcome of off-PN.

After 6 months or more of surgery, although long-term decrease of eGFR and percent eGFR in both groups was found in the included studies, the pooled meta-analysis for postoperative long-term eGFR change and percent eGFR change revealed a significantly greater decrease in the on-PN group than in the off-PN group. Between-study heterogeneity was significant for long-term eGFR change, the random-effects model was then utilized to reduce the effect of heterogeneity, whereas the long-term percent eGFR change was only the opposite. Subgroup analysis for both long-term eGFR change and percent eGFR change showed that the included large sample studies [22, 25, 30–33] and small sample studies [9, 19, 29, 36, 38] was pooled without significant between-study heterogeneity. The same conclusions were drawn in a large-sample subgroup with original pooled analysis, whereas no significant difference was found between two groups in the small sample subgroup. Sample size was considered a reason for heterogeneity, and a large sample subgroup is believed to be close to the truth. Sensitivity analysis was performed by exclusion of the highest scored study or studies in the modified Newcastle−Ottawa Scale. No change in the significance of the outcome was noted.

No significant difference was found between two groups for postoperative long-term Cr change. The random-effects model was used to pool the included studies owing to the significant difference of between-study heterogeneity. We attributed this result to the limited number of included studies. Sensitivity analysis with exclusion of the highest scored study [38] found a significant difference between the two groups about long-term Cr change and a very low degree of between-study heterogeneity. Additional studies are needed to confirm the conclusion.

The pooled data of postoperative increase in CKD (stage≥3) suggests the lack of significant difference between the two groups, and the degree of between-study heterogeneity was moderate. In fact, eGFR after on-PN may not decrease to 60 ml/min/1.73 m^2^ or lower even when renal ischemia−reperfusion injury truly exists.

The ARF rate is significantly lower for the off-PN group than for the on-PN group. This result may be explained by the injury caused by renal ischemia during operation.

To assess any impact of the highest scored study or studies on the effect on primary outcomes, we performed a sensitivity analysis with exclusion of the highest scored study or studies. Given the moderate degree of between-study heterogeneity, all results performed with the fixed-effects model were similar to those of the original analysis except for the long-term Cr change, which was significantly increased in the on-PN group than in the off-PN group (WMD: − 0.09; 95%CI: − 0.13 to − 0.04; *p* = 0.0002).

### Limitations and strength

The primary limitation of this systematic review and meta-analysis was that no RCTs were included for evaluation and subsequent analysis; hence, sufficient data are difficult to acquire for meaningful results. Moreover, the studies that provide data on the change in postoperative long-term Cr level were exceedingly few to offer a more convincing result than currently attained. In addition, the operations were performed by surgeons with different levels of surgical expertise and different choices of surgical approaches. Finally, patient allocation and treatment assignment were usually based on the physician’s attitude instead of randomized allocation; this aspect led to a significant selection bias.

Although a small number of papers have compared the two surgical procedures, no paper has discussed their long-term impact on renal function; this topic is particularly important to effectively choose the proper treatment when possible. An increasing number of T1-T2a peripheral renal tumors have been detected at a young age, and an improved outcome is meaningful. This meta-analysis was conducted at an appropriate time. A sufficient number of studies have been accumulated for inspection by meta-analytical methods. Studies were identified using multiple strategies; the methodological quality of the studies was evaluated on the basis of strict inclusion and exclusion. Subgroup and sensitivity analyses were performed to analyze the source of heterogeneity. The MOOSE guidelines were used to report our systematic review. Publication bias was not significant.

## Conclusions

This meta-analysis demonstrated that off-PN positively impacts short- and long-term renal function relative to that of conventional on-PN. Given the inherent limitations of included studies, large-volume and well-designed RCTS with extensive follow up are wanted to confirm and update the conclusion of this analysis in future.

## Additional files

Additional file 1: **Table S1.** Risk of bias in included studies using modified Newcastle-Ottawa Scale. (DOCX 51 kb)

Additional file 2: **Figure S1.** Forest plot and meta-analysis of postoperative short-term eGFR. (PDF 83 kb)

Additional file 3: **Figure S2.** Forest plot and meta-analysis of postoperative short-term Cr level. (PDF 83 kb)

Additional file 4: **Figure S3.** Forest plot and meta-analysis of postoperative long-term eGFR. (PDF 160 kb)

Additional file 5: **Figure S4.** Forest plot and meta-analysis of postoperative acute renal failure. (PDF 159 kb)

Additional file 6: **Figure S5.** Forest plot and meta-analysis of postoperative newly increased chronic kidney disease (CKD)(stage≥3). (PDF 82 kb)

Additional file 7: **Figure S6.** Forest plot and subgroup meta-analysis of postoperative short-term eGFR change. (PDF 84 kb)

Additional file 8: **Figure S7.** Forest plot and subgroup meta-analysis of postoperative short-term Cr level change. (PDF 84 kb)

Additional file 9: **Figure S8.** Forest plot and subgroup meta-analysis of postoperative long-term eGFR change. (PDF 84 kb)

Additional file 10: **Figure S9.** Forest plot and subgroup meta-analysis of postoperative long-term % eGFR change. (PDF 84 kb)

Additional file 11: **Figure S10.** Forest plot and subgroup meta-analysis of postoperative long-term change of Cr level. (PDF 83 kb)

## Abbreviations

ARF: Acute renal failure
CI: Confidence interval
CKD: Chronic kidney disease
Cr: Creatinine
eGFR: estimated glomerular filtration rate
off-PN: off-clamp partial nephrectomy
on-PN: on-clamp partial nephrectomy
OR: Odds ratio
WMD: Weighted mean difference
