# Supplementary material for: Off-clamp partial nephrectomy has a positive impact on short- and long-term renal function: a systematic review and meta-analysis
Source: BMC Nephrol. 2018 Jul 31;19:188. doi: 10.1186/s12882-018-0993-3 (PMC6069776; doi:10.1186/s12882-018-0993-3)
Supplement: Supplementary file 1 — Table S1. Risk of bias in included studies using modified Newcastle-Ottawa Scale. (DOCX 51 kb) [file 12882_2018_993_MOESM1_ESM.docx]

**Additional table. Risk of bias in included studies using modified Newcastle-Ottawa Scale**

| **Table S1.** **Modified Newcastle-Ottawa Scale** | | | | |
| --- | --- | --- | --- | --- |
| **Study** | **Selection**  Assignment Representative Representative  for treatment cohort group reference group | **Comparability**  Comparable for  1,2,3,4,5,6,7,8,9 | **Outcome**  Assessment of Adequate  outcome fellow-up | **Quality**  **Score** |
| Guillonneau^2003[^[^16^](#_ENREF_16)^]^  Kane^2004[^[^17^](#_ENREF_17)^]^  Kobayashi^2008[^[^18^](#_ENREF_18)^]^  Hong^2009[^[^20^](#_ENREF_20)^]^  Koo^2010[^[^21^](#_ENREF_21)^]^  Thompson^2010[^[^22^](#_ENREF_22)^]^  Smith^2011[^[^23^](#_ENREF_23)^]^  Petrasz^2012[^[^24^](#_ENREF_24)^]^  George^2013[^[^26^](#_ENREF_26)^]^  Kaczmarek^2013[^[^28^](#_ENREF_28)^]^  Porpiglia^2012[^[^25^](#_ENREF_25)^]^  Salevitz^2015[^[^34^](#_ENREF_34)^]^  Ener^2016[^[^35^](#_ENREF_35)^]^  Wang^2016[^[^36^](#_ENREF_36)^]^  Anderson^2017[^[^37^](#_ENREF_37)^]^  Rosen^2017[^[^38^](#_ENREF_38)^]^  Verze^2017[^[^39^](#_ENREF_39)^]^  Weizer^2008[^[^19^](#_ENREF_19)^]^  Hung^2013[^[^27^](#_ENREF_27)^]^  Lee^2014[^[^32^](#_ENREF_32)^]^  Komninos^2015[^[^33^](#_ENREF_33)^]^  Taweemonkongsap^2018[^[^9^](#_ENREF_9)^]^  Acar^2014[^[^30^](#_ENREF_30)^]^  Krane^2013[^[^29^](#_ENREF_29)^]^  Jabaji^2014[^[^31^](#_ENREF_31)^]^ | no yes yes  no yes yes  no yes yes  no yes yes  no yes yes  no yes yes  no yes yes  no yes yes  no yes yes  no yes yes  no yes yes  no yes yes  no yes yes  no yes yes  no yes yes  no yes yes  no yes yes  no yes yes  no yes yes  no yes yes  no yes yes  no yes yes  no yes yes  no yes yes  no yes yes | 1,2,3 ,7  1,2,3,4 ,7  1,2,7  1,2,5,6,7,9  1,2,7  1,5  1,3,6  1,2  1,3,4,5,7,9  1,3,4,5,7  1,3,4,5,6,7,8  3,5,7  1,2,3,5,7,8  1,2,3,5,6,7,8,9  1,2,3,6,7,8  1,2,3,4,5,6,7,8,9  1,2,3,5,6,7,8  NA  NA  NA  NA  NA  1,2,3,4 ,5,6,8  NA  NA | creatinine yes  creatinine yes  creatinine yes  GFR yes  creatinine unclear  GFR yes  creatinine,eGFR yes  creatinine unclear  creatinine,eGFR yes  GFR yes  creatinine,eGFR yes  creatinine,eGFR yes  creatinine,eGFR yes  eGFR yes  eGFR yes  eGFR yes  creatinine,eGFR yes  creatinine yes  creatinine,eGFR yes  eGFR yes  creatinine,eGFR yes  eGFR yes  eGFR yes  creatinine,GFR yes  eGFR yes | 6  6  5  6  5  5  5  4  6  6  7  5  6  8  6  8  7  4  4  4  4  4  7  4  4 |
| Comparability variables:1=age,2=tumor size,3= preoperative renal function,4= American Society of Anesthesiologists score,5=gender,6=tumor side,7=body mass index,8=nephrometry(R.E.N.A.L) Score,9=tumor location | | | | |
