# Supplementary figures and images for: Off-clamp partial nephrectomy has a positive impact on short- and long-term renal function: a systematic review and meta-analysis
Source: BMC Nephrol. 2018 Jul 31;19:188. doi: 10.1186/s12882-018-0993-3 (PMC6069776; doi:10.1186/s12882-018-0993-3)

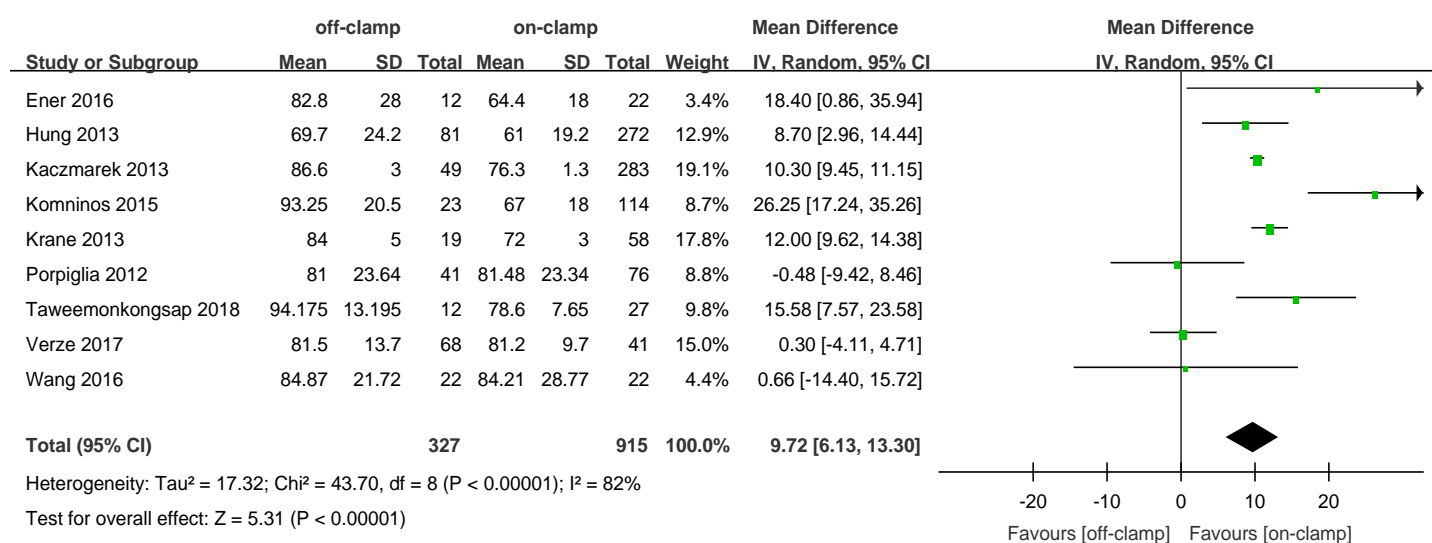

Supplement: Supplementary file 2 — Figure S1. Forest plot and meta-analysis of postoperative short-term eGFR. (PDF 83 kb) [file 12882_2018_993_MOESM2_ESM.pdf]

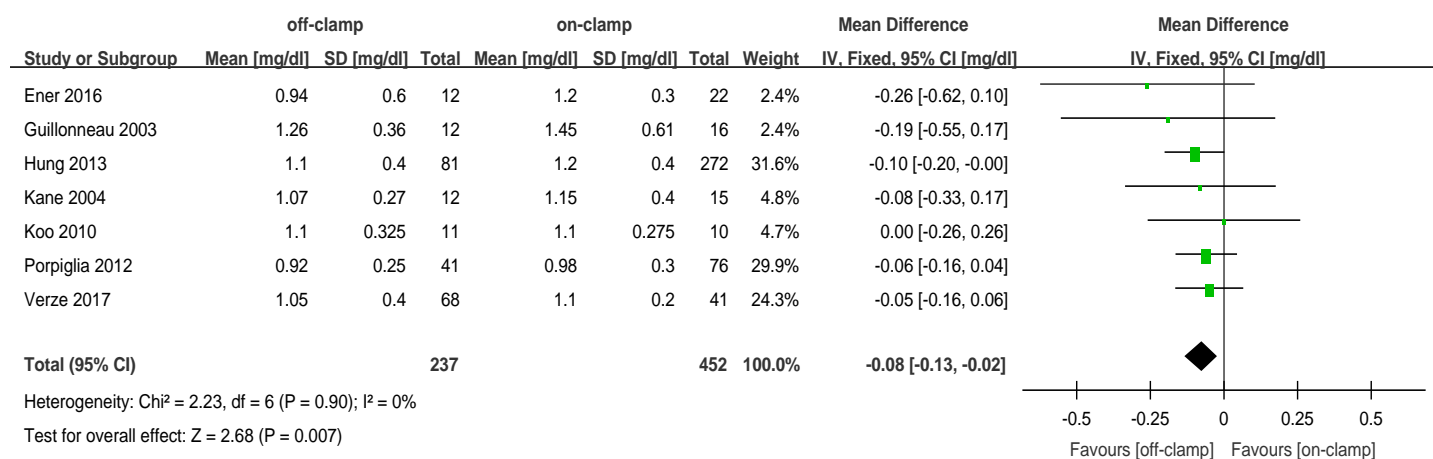

Supplement: Supplementary file 3 — Figure S2. Forest plot and meta-analysis of postoperative short-term Cr level. (PDF 83 kb) [file 12882_2018_993_MOESM3_ESM.pdf]

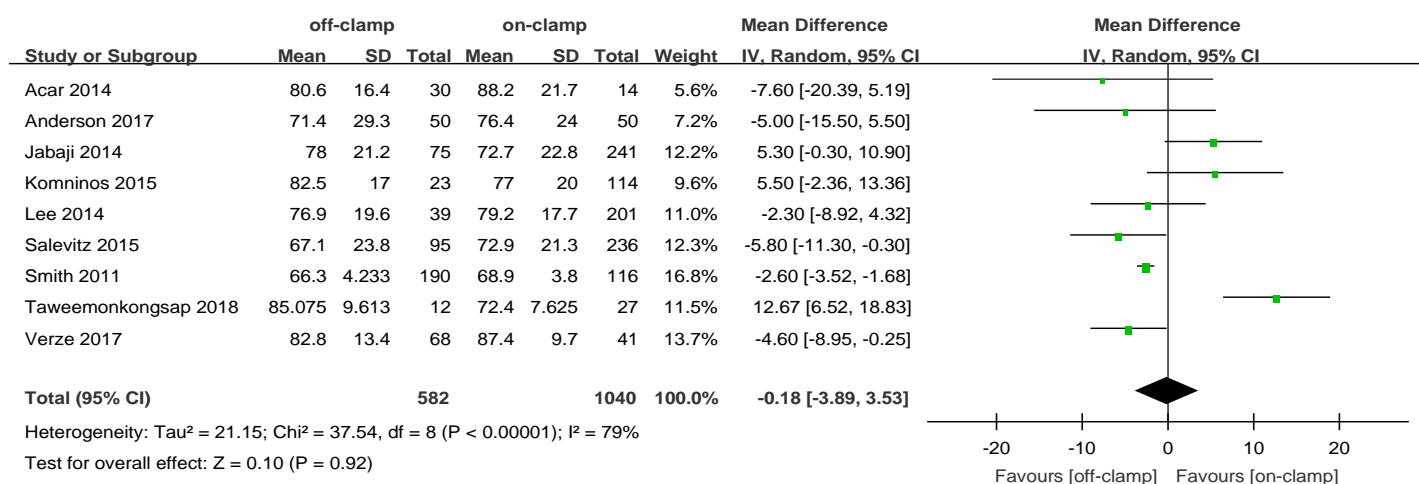

Supplement: Supplementary file 4 — Figure S3. Forest plot and meta-analysis of postoperative long-term eGFR. (PDF 160 kb) [file 12882_2018_993_MOESM4_ESM.pdf]

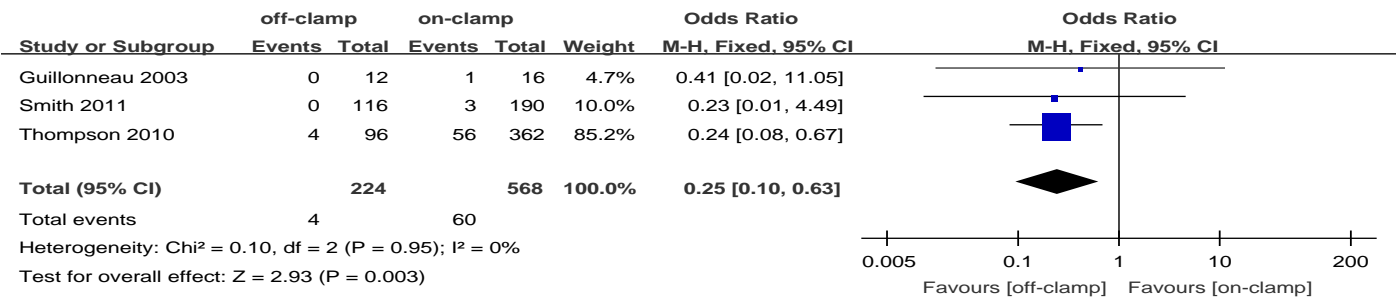

Supplement: Supplementary file 5 — Figure S4. Forest plot and meta-analysis of postoperative acute renal failure. (PDF 159 kb) [file 12882_2018_993_MOESM5_ESM.pdf]

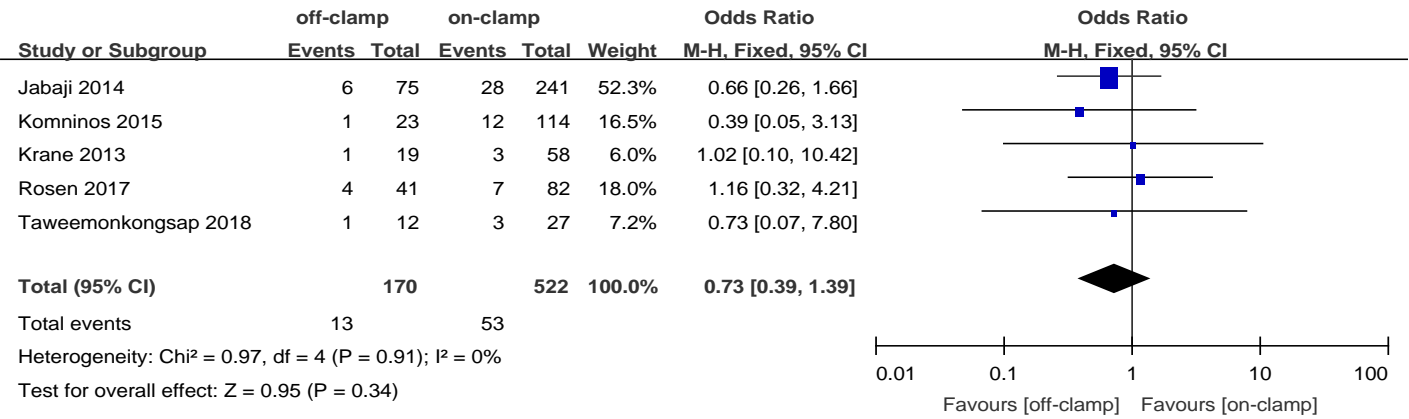

Supplement: Supplementary file 6 — Figure S5. Forest plot and meta-analysis of postoperative newly increased chronic kidney disease (CKD)(stage≥3). (PDF 82 kb) [file 12882_2018_993_MOESM6_ESM.pdf]

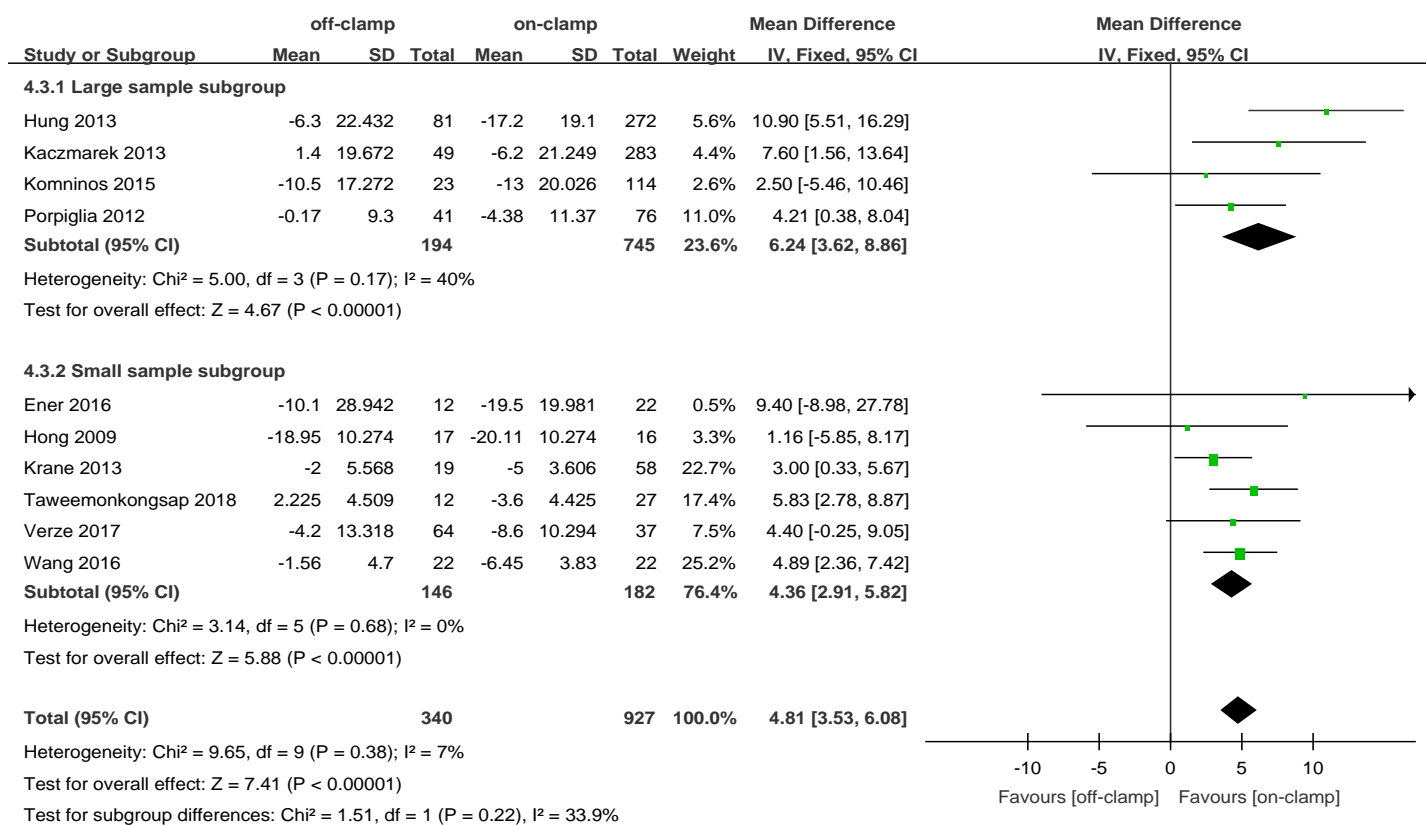

Supplement: Supplementary file 7 — Figure S6. Forest plot and subgroup meta-analysis of postoperative short-term eGFR change. (PDF 84 kb) [file 12882_2018_993_MOESM7_ESM.pdf]

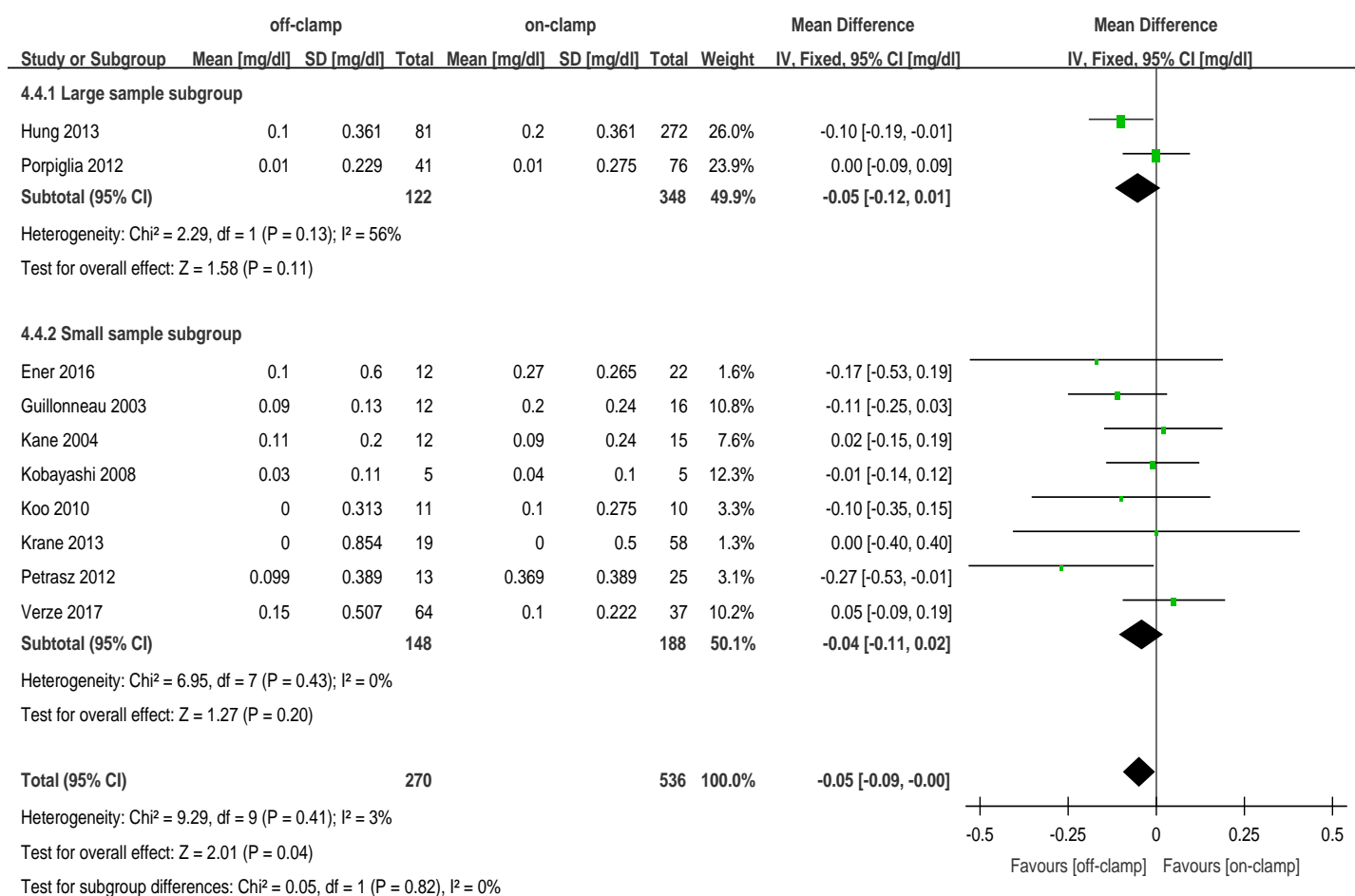

Supplement: Supplementary file 8 — Figure S7. Forest plot and subgroup meta-analysis of postoperative short-term Cr level change. (PDF 84 kb) [file 12882_2018_993_MOESM8_ESM.pdf]

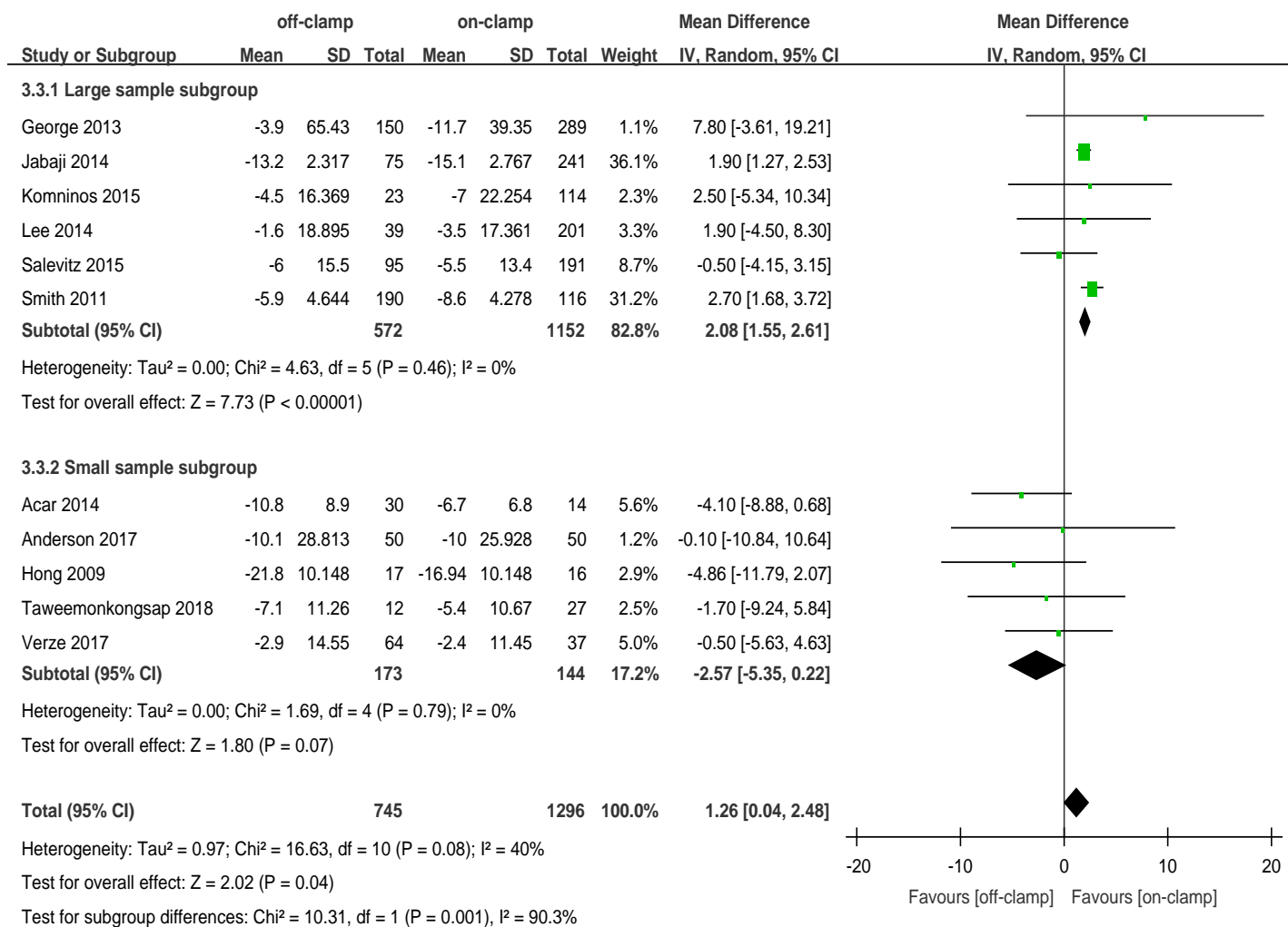

Supplement: Supplementary file 9 — Figure S8. Forest plot and subgroup meta-analysis of postoperative long-term eGFR change. (PDF 84 kb) [file 12882_2018_993_MOESM9_ESM.pdf]

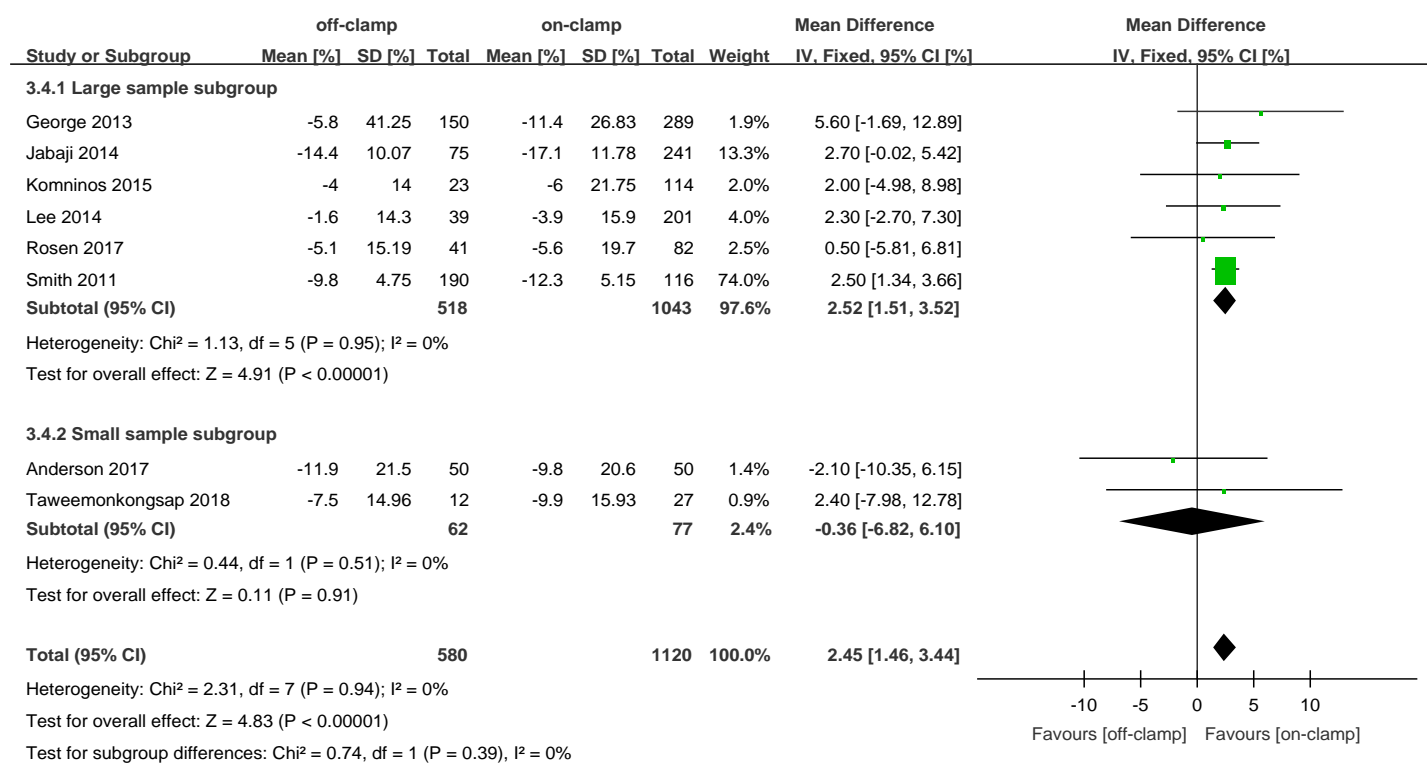

Supplement: Supplementary file 10 — Figure S9. Forest plot and subgroup meta-analysis of postoperative long-term % eGFR change. (PDF 84 kb) [file 12882_2018_993_MOESM10_ESM.pdf]

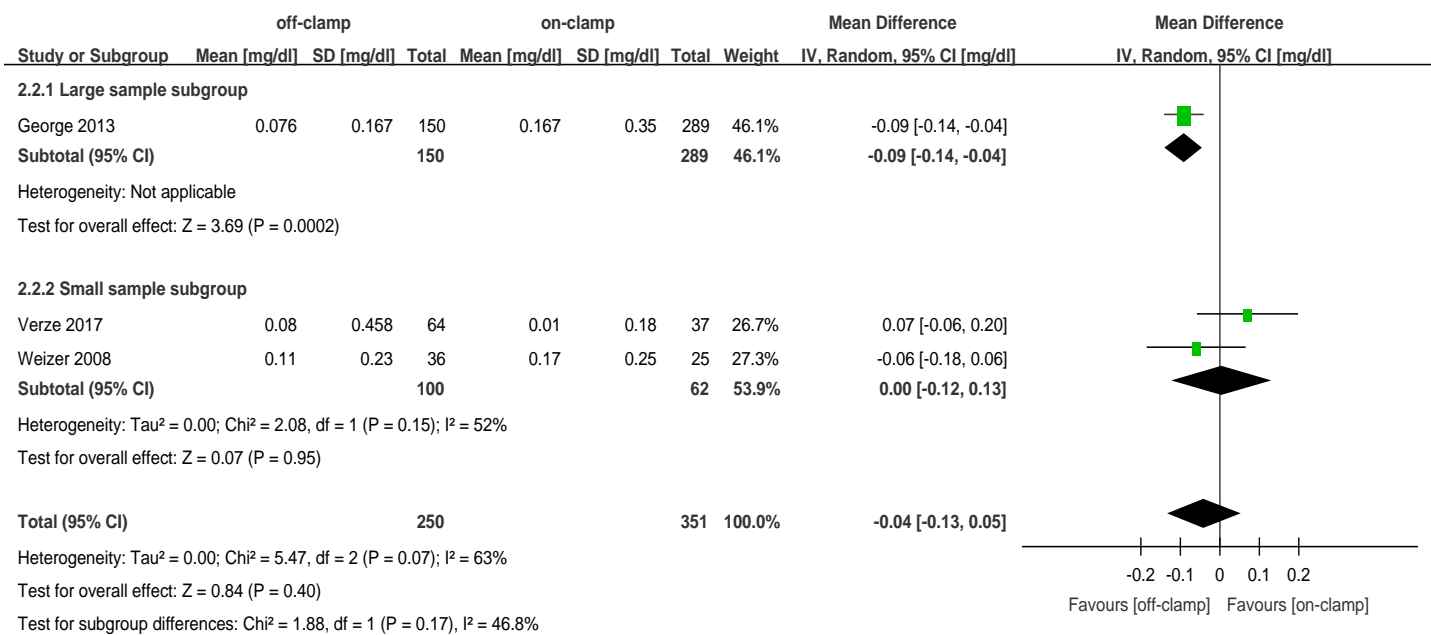

Supplement: Supplementary file 11 — Figure S10. Forest plot and subgroup meta-analysis of postoperative long-term change of Cr level. (PDF 83 kb) [file 12882_2018_993_MOESM11_ESM.pdf]
